# The multivariate physical activity signatures associated with body mass index and waist-to-height ratio in 3–5-year-old Norwegian children

**DOI:** 10.1016/j.pmedr.2022.101930

**Published:** 2022-07-29

**Authors:** Eivind Aadland, Ada Kristine Ofrim Nilsen, Elisabeth Straume Haugland, Kristoffer Buene Vabø, Katrine Nyvoll Aadland

**Affiliations:** Western Norway University of Applied Sciences, Faculty of Education, Arts and Sports, Department of Sport, Food and Natural Sciences, Campus Sogndal, Sogndal, Norway

**Keywords:** Children, Preschoolers, Accelerometer, Adiposity, Multivariate pattern analysis

## Abstract

The evidence regarding associations between intensity-specific physical activity and adiposity in young children is inconclusive. The aim of this study was to determine the multivariate physical activity intensity signatures associated with body mass index and waist-to-height ratio in children aged 3–5 years. A sample of 1003 Norwegian preschool children (mean age 4.3 years, 51 % boys) from the Active Learning Norwegian Preschool(er)s study provided data on physical activity (ActiGraph GT3X+), body mass index, and waist-to-height ratio during 2019–2020. Multivariate pattern analysis was used to determine associations between the triaxial intensity spectrum (0–99 to ≥15,000 counts per minute) and the outcomes. We found significant associations for physical activity with body mass index and waist-to-height ratio (explained variances = 10.8 and 11.5 %, respectively). For the vertical axis, associations were negative for time spent sedentary (0–99 counts per minute) and positive for time spent in lower intensities (100–2999 counts per minute) for both outcomes, whereas associations for vigorous intensities (≥4000 counts per minute) differed for body mass index (no associations) and waist-to-height ratio (negative associations). Association patterns for body mass index and waist-to-height ratio were rather similar, but the stronger associations with vigorous physical activity intensities for waist-to-height ratio suggest that waist-to-height ratio might better capture adiposity resulting from inactivity than body mass index.

## Highlights

• Physical activity intensities are associated with body size in young children.
• Associations are comparable for body mass index and waist-to-height ratio.
• Associations are stronger for triaxial than for uniaxial accelerometer data.

## 1 Introduction

Overweight and obesity, which are major health concerns globally (The Global Burden of Disease 2015 Obesity Collaborators), often develop from an early age (Monteiro and Victora, 2005). There is convincing evidence of a negative association for physical activity (PA) with adiposity and metabolic health in school-aged children and youth (Poitras et al., 2016, Ekelund et al., 2012, Aadland et al., 2018, Jimenez-Pavon et al., 2010, Cooper et al., 2015). However, as the evidence for such associations in younger children is inconclusive (Cooper et al., 2015, Carson et al., 2017, Bingham et al., 2016, Wiersma et al., 2020), it is uncertain when these relationships start to emerge.

A recent systematic review and meta-analysis by Wiersma et al. (2020) showed no associations between PA and body mass index (BMI) (15 studies) or waist circumference (5 studies) in preschool-aged children, except a *positive* association between moderate to vigorous intensity PA (MVPA) and BMI in boys. Interestingly, sedentary time (SED) was *negatively* associated with BMI. On the contrary, SED was not associated and both MVPA and vigorous intensity PA (VPA) were negatively associated with weight status (normal weight versus overweight or obese as defined by BMI) (19 studies). While the lack of significant associations between PA and BMI generally is consistent with other systematic reviews (Bingham et al., 2016, Carson et al., 2017), the negative association with weight status contrasts the null finding in the International Children’s Accelerometry Database (Cooper et al., 2015).

The mixed and partly counterintuitive findings by Wiersma (Wiersma et al., 2020) might result from the inclusion of studies with small to moderate sample sizes (n = 46–540), inconsistencies in accelerometer data reduction methods across studies, and the inclusion of only few PA intensities in the analyses in most studies. To obtain a better and more detailed picture of how PA associates with adiposity, studies should include the entire PA intensity spectrum in their analysis (Poitras et al., 2016, Wiersma et al., 2020, van der Ploeg and Hillsdon, 2017, Pedisic, 2014), derived from a harmonized data reduction approach. To solve these challenges, Aadland et al (Aadland et al., 2021) used multivariate pattern analysis to determine the association pattern across the full triaxial PA intensity spectrum with BMI and weight status in 1182 preschoolers. Multivariate pattern analysis is widely applied in other fields of research with the objective of revealing patterns of important biomarkers among many highly interrelated variables (Rajalahti and Kvalheim, 2011, Rajalahti et al., 2010, Madsen et al., 2010), and was introduced as a means for analyzing PA intensity spectrum data from accelerometry by Aadland et al in 2018 (Aadland et al., 2018, Aadland et al., 2020). Since this method allows for combining all PA intensities - irrespective of the number of variables and multicollinearity among them - in a joint regression model (Wold et al., 1984, Kvalheim and Karstang, 1989), it is a powerful approach to determine complete PA intensity association signatures with outcomes (Aadland et al., 2018, Aadland et al., 2021, Aadland et al., 2019). Aadland et al (Aadland et al., 2021) confirmed a negative association between SED and BMI in preschoolers, as shown by Wiersma et al (Wiersma et al., 2020), but found positive associations for light intensity PA (LPA) and negative associations for VPA. Associations for VPA were not evident in the youngest children but emerged in children aged 5–6 years.

The main aim of the present study was to verify the findings on associations between PA and BMI shown by Aadland et al (Aadland et al., 2021) in a new, large sample of preschoolers using the same multivariate analytic approach. In addition, since waist-to-height ratio (WHtR) has been suggested as a useful indicator of cardiometabolic risk in children (Jiang et al., 2021, Ezzatvar et al., 2021), but few studies have determined associations between PA and waist circumference and/or WHtR in preschoolers (Wiersma et al., 2020), we aimed to investigate potential differences in association patterns with PA for BMI and WHtR. Despite waist circumference and WHtR appears to better capture abdominal adiposity than BMI, their performance as markers of cardiometabolic risk in children has been shown to be similar (Reilly et al., 2010). Thus, we determined the multivariate PA intensity signatures associated with BMI and WHtR in a large sample of children aged 3–5 years and hypothesized that association patterns would be similar for both outcomes.

## 2 Subjects and methods

The present cross-sectional study used baseline data from the Active Learning Norwegian Preschool(er)s (ACTNOW) study (Aadland et al., 2020). ACTNOW is a cluster randomized controlled trial investigating the effect of a multicomponent preschool PA intervention in Sogn og Fjordane County, a rural area in western Norway, conducted between September 2019 and June 2022. A total of 1265 preschool children aged 2.7–6.6 years (born in 2014–2017) from 46 preschools (response rate of preschools: 82 %; response rate of children: 83 %) participated in the study.

Parents of all participating children received oral and written information about the study and provided written consent prior to testing. Preschools received information and agreed to participate in the study. We explained the procedures according to the children’s level of understanding. The institutional ethics committee and the Norwegian Centre for Research Data (NSD) approved the study (reference number 248220). The study is registered in clinicaltrials.gov August 7, 2019, with identifier NCT04048967 (https://clinicaltrials.gov/ct2/show/NCT04048967?term=actnow&rank=1).

## 3 Procedures

### 3.1 Physical activity measurement

PA was measured using ActiGraph GT3X+ accelerometers (ActiGraph, LLC, Pensacola, Florida, USA) (John and Freedson, 2012). Children wore the accelerometer in an elastic belt on the right hip and were instructed to wear the monitor at all times for 7 consecutive days, except during water-based activities. Units were initialized at a sampling rate of 30 Hz and files were analyzed restricted to hours 06:00 to 21:59 using MATLAB. We used 1-second epoch to avoid misclassification of PA intensities, in particular to capture high intensity PA correctly (Aadland et al., 2020, Aadland and Nilsen, 2022). Periods of ≥20 min of zero counts were defined as non-wear time (Esliger et al., 2005). We applied wear time requirements of ≥8 h/day and ≥3 weekdays and ≥1 weekend day to constitute a valid measurement (Aadland et al., 2020).

We have previously applied 2 different resolutions of PA intensity spectra in multivariate pattern analyses in preschoolers (17 and 33 intensity variables from 0–99 to ≥15,000 counts per minute [cpm]) (Aadland et al., 2021, Nilsen et al., 2020) and have found similar model fit when using motor skills as the outcome (Aadland et al., 2020). In the present study, we compared the performance of both resolutions for both outcomes, showing comparable model fit and interpretation (explained variances (R^2^) = 12.4 versus 10.8 % for BMI and 13.1 versus 11.5 % for WHtR). Thus, we used the simpler descriptor of 17 variables in all analyses.

We created a dataset with 17 intensity variables to capture movement in narrow intensity intervals across the triaxial intensity spectra, from 0–99, 100–999, 1000–1999, 2000–2999, … 14,000–14,999, to ≥15,000 cpm, from each of the three axes (i.e., the vertical, anteroposterior, and mediolateral axes) and the vector magnitude. Thus, every second of the valid wear time were classified as being within one of these 17 intensity bins to obtain detailed information on children’s intensity distribution. For descriptive purposes, we reported total PA (average cpm; a measure of total movement over the measurement period), and minutes per day spent SED (≤100 cpm), in LPA (101–2295 cpm), MPA (2296–4011 cpm), VPA (≥4012 cpm), and in MVPA (min/day) (≥2296 cpm) using the cut points suggested by Evenson et al (Evenson et al., 2008) applied to the vertical axis. The reporting of associations was primarily based on the vertical axis and these cut points were used to guide our interpretation of associations for the intensity spectrum post hoc.

### 3.2 Anthropometrics and demographics

Body mass was measured to the nearest 0.1 kg using an electronic scale (Seca 899, SECA GmbH, Hamburg, Germany), and height was measured to the nearest 0.1 cm with a portable stadiometer (Seca 217, SECA GmbH). BMI (kg/m^2^) was calculated, and children were classified as normal weight (including underweight), overweight, or obese based on criteria proposed by Cole et al. (2000). Waist circumference was measured twice to the nearest 0.5 cm at the level of the umbilical zone using a circumference measuring tape (Seca 201, GmbH). WHtR (cm/cm) was calculated. Parental socioeconomic status (based on the highest education level of mother or father) was assessed using a questionnaire completed by each child’s mother and/or father at baseline.

### 3.3 Statistical analysis

Children’s characteristics and PA were reported as frequencies, means, and standard deviations (SDs). The multivariate PA intensity signatures associated with outcomes were determined using multivariate pattern analysis applied to the triaxial intensity spectrum, equivalent to its previous application to accelerometer data (Nilsen et al., 2020, Aadland et al., 2018, Aadland et al., 2021). Partial least squares (PLS) regression analyses (Wold et al., 1984) were used to determine the association pattern between outcomes and the PA intensity spectrum. The primary analysis included all 51 variables from the 3 axes as explanatory variables in one joint model. The uniaxial (vertical axis) and the vector magnitude intensity spectra were analyzed separately in secondary analyses. PLS regression handles completely collinear variables through decomposing the explanatory variables into orthogonal linear combinations (PLS components), while simultaneously maximizing the covariance with the outcome variable (Wold et al., 1984). Models with different number of components were cross-validated using Monte Carlo resampling (Kvalheim et al., 2018) with 1000 repetitions by repeatedly and randomly keeping 50 % of the subjects as an external validation set. We retained the model with the number of PLS components leading to the lowest prediction error. For each model, we used target projection (Kvalheim and Karstang, 1989, Rajalahti and Kvalheim, 2011) followed by reporting of multivariate correlation coefficients with 95 % confidence intervals (CIs) to show the importance of each PA intensity variable in the multivariate space (Rajalahti et al., 2009, Rajalahti et al., 2009, Aadland et al., 2019). To adjust for sources of variation and confounding, we obtained residuals from linear regression models using BMI (adjusted for sex and age), WHtR (adjusted for sex and age) and PA variables (adjusted for sex, age, and wear time) as outcomes, prior to performing the multivariate pattern analysis. We compared the association patterns between boys and girls and between younger and older children (median split by age) by performing the analyses separately for these subgroups. The association patterns were compared among groups by correlating association patterns using Pearson’s r. Formal testing of moderators was not performed because such analyses are difficult to perform and challenging to interpret for multivariate association patterns including a spectrum of many explanatory variables. Multivariate pattern analyses were performed using the commercial software Sirius version 11.5 (Pattern Recognition Systems AS, Bergen, Norway).

## 4 Results

### 4.1 Children’s characteristics

Of the 1265 children that participated in the study, 1003 (79 %) children provided valid data for PA, BMI, and WHtR and were included in the present analysis. The included children did not differ from the excluded children on explanatory (p ≥0.255) or outcome (p ≥0.117) variables. The children’s characteristics are shown in Table 1. The age range of the younger and older children were 2.7–4.3 and 4.4–6.6 years, respectively.

**Table 1:** Children’s characteristics. Values are means (SDs) if not otherwise stated.

	**Total**	**Boys**	**Girls**	**Younger**	**Older**
n	1003	516	487	494	509
Age (years)	4.3 (0.9)	4.3 (0.9)	4.4 (0.9)	3.6 (0.5)	5.1 (0.4)
Body mass (kg)	18.5 (3.2)	18.6 (3.2)	18.4 (3.3)	16.6 (2.2)	20.3 (3.0)
Height (cm)	106 (8)	107 (8)	106 (8)	101 (5)	112 (5)
Body mass index (kg/m^2^)	16.3 (1.5)	16.3 (1.4)	16.3 (1.6)	16.4 (1.4)	16.2 (1.5)
Overweight/Obese (%)^1^	14.6/2.8	11.0/2.3	18.3/3.3	13.0/2.2	16.1/3.3
Waist circumference (cm)	53.2 (4.3)	53.1 (4.1)	53.4 (4.5)	51.8 (3.7)	54.6 (4.4)
Waist-to-height ratio (cm/cm)	0.50 (0.04)	0.50 (0.04)	0.51 (0.04)	0.52 (0.04)	0.49 (0.04)
Parental education level (%)^2^					
Upper secondary school	24.8	26.1	23.4	25.1	24.5
University <4 years	28.6	30.3	26.8	29.1	28.1
University ≥4 years	46.6	43.6	49.8	45.8	47.4
Physical activity^3^					
Wear days (n)	6.9 (0.9)	6.9 (0.9)	6.9 (1.0)	7.0 (0.9)	6.9 (1.0)
Wear time (min/day)	763 (69)	768 (69)	758 (69)	758 (68)	768 (70)
Total activity (cpm)	684 (153)	709 (153)	657 (149)	653 (141)	715 (158)
SED (min/day)	539 (64)	534 (64)	544 (65)	533 (62)	543 (66)
LPA (min/day)	150 (21)	156 (21)	144 (20)	153 (21)	147 (22)
MPA (min/day)	38 (8)	40 (8)	35 (7)	36 (7)	39 (8)
VPA (min/day)	37 (10)	39 (11)	35 (10)	34 (9)	40 (10)
MVPA (min/day)	74 (17)	79 (17)	70 (15)	70 (16)	78 (17)
Achieved guideline (%)^4^	81.1	86.2	75.6	74.5	87.4

SED = sedentary time; LPA = light physical activity; MPA = moderate physical activity; VPA = vigorous physical activity; MVPA = moderate-to-vigorous physical activity. ^1^The proportion being overweight or obese defined by the Cole et al. (2000) criteria. ^2^Data on parental education was available for n = 948 children. ^3^Defined by the Evenson et al cut points (Evenson et al., 2008) applied to the vertical axis. ^4^Defined as a mean of 60 min MVPA/day.

### 4.2 Association patterns

**Fig. 1:**
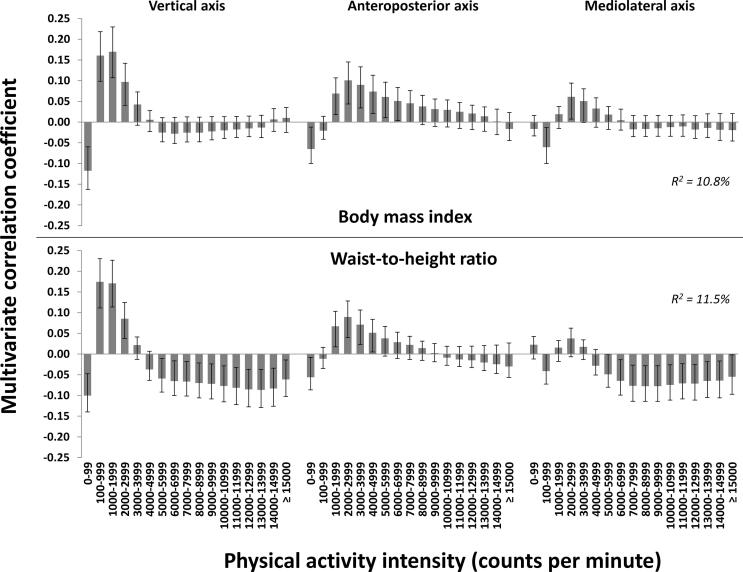
The multivariate physical activity signatures associated with body mass index and waist-to-height ratio in preschoolers. Results are reported as multivariate correlation coefficients from a joint model including 51 physical activity intensity variables from triaxial accelerometry. Multivariate correlation coefficients can be interpreted equivalent to bivariate correlations, though they are derived from the multivariate model.

The multivariate triaxial PA signatures associated with BMI (R^2^ = 10.8 %, 5 PLS components) and WHtR (R^2^ = 11.5 %, 5 PLS components) in the total sample of children are shown in Fig. 1. The association patterns were similar for both outcomes (r = 0.94). For BMI, associations for the vertical axis were negative for time spent in 0–99 cpm, positive for time spent in 100–2999 cpm, and not significant for time spent ≥3000 cpm. For WHtR, associations for the vertical axis were negative for time spent in 0–99 cpm, positive for time spent in 100–2999 cpm, and negative for time spent ≥5000 cpm. The association patterns shown for the vertical axis were partly reflected by the anteroposterior and mediolateral axes. Across outcomes, the use of triaxial data resulted in approximately two- to threefold higher explained variances than uniaxial (vertical axis) (R^2^ = 4.6 % for BMI and 6.2 % for WHtR) and vector magnitude data (R^2^ = 4.2 % for BMI and 4.3 % for WHtR). Association patterns for the vertical axis were similar for both outcomes when using uniaxial and triaxial data (r = 0.98). The association patterns using vector magnitude data are shown in Supplemental Fig. 1.

**Fig. 2:**
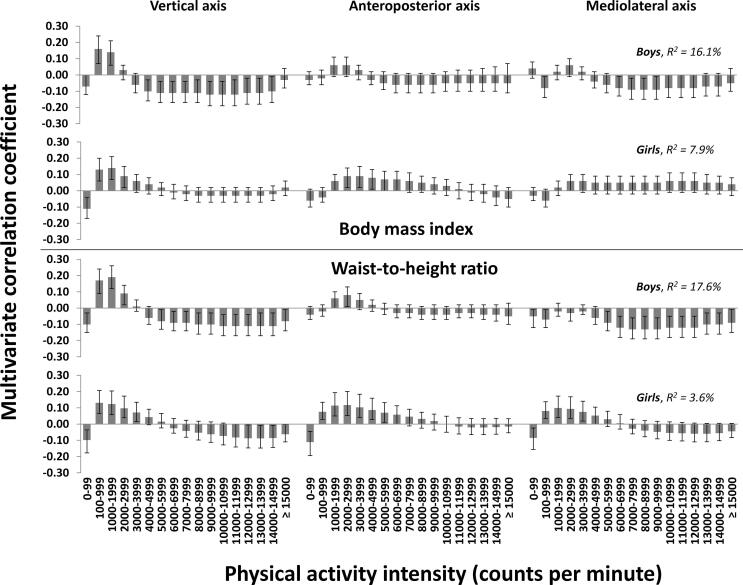
The multivariate physical activity signatures associated with body mass index and waist-to-height ratio in boys and girls. Results are reported as multivariate correlation coefficients from a joint model including 51 physical activity intensity variables from triaxial accelerometry. Multivariate correlation coefficients can be interpreted equivalent to bivariate correlations, though they are derived from the multivariate model.

Associations were stronger in boys (R^2^ = 16.1 %, 5 PLS components for BMI; R^2^ = 17.6 %, 5 PLS components for WHtR) than in girls (R^2^ = 7.9 %, 4 PLS components for BMI; R^2^ = 3.6 %, 2 PLS components for WHtR) for both outcomes (Fig. 2). In boys, associations for the vertical axis were negative for time spent in 0–99 cpm (BMI and WHtR), positive for time spent in 100–1999 cpm (BMI)/100–2999 (WHtR), and negative for time spent ≥4000 cpm (BMI)/≥5000 cpm (WHtR). In girls, associations for the vertical axis were negative for time spent in 0–99 cpm (BMI and WHtR), positive for time spent in 100–2999 cpm (BMI)/100–3999 (WHtR), and not significant for time spent ≥4000 cpm (BMI)/negative for time spent ≥12,000 cpm (WHtR).

**Fig. 3:**
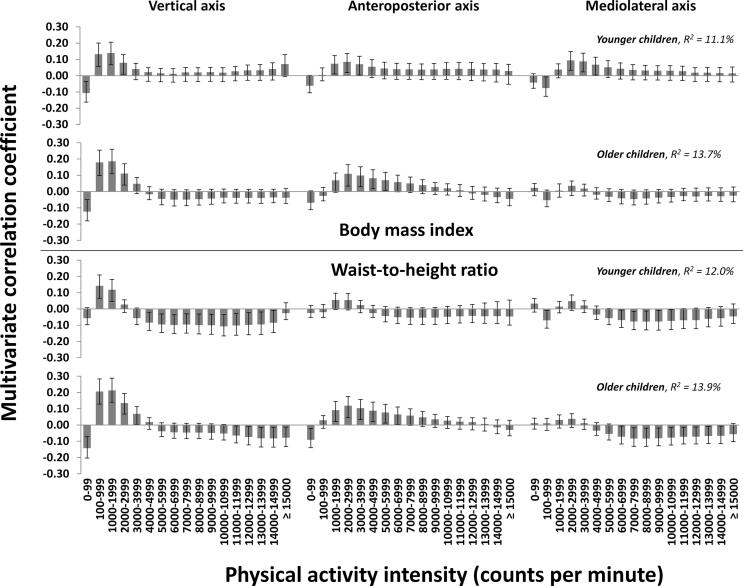
The multivariate physical activity signatures associated with body mass index and waist-to-height ratio in younger (2.7–4.3-year-old) and older (4.4–6.6-year-old) children. Results are reported as multivariate correlation coefficients from a joint model including 51 physical activity intensity variables from triaxial accelerometry. Multivariate correlation coefficients can be interpreted equivalent to bivariate correlations, though they are derived from the multivariate model.

Associations were marginally stronger in older children (R^2^ = 13.7 %, 5 PLS components for BMI; R^2^ = 13.9 %, 5 PLS components for WHtR) than in younger children (R^2^ = 11.1 %, 5 PLS components for BMI; R^2^ = 12.0 %, 5 PLS components for WHtR) for both outcomes (Fig. 3). For BMI, associations for the vertical axis were negative for time spent in 0–99 cpm, positive for time spent in 100–2999 cpm and not significant for time spent ≥4000 cpm in both age groups. For WHtR, associations for the vertical axis were not significant for time spent in 0–99 cpm, positive for time spent in 100–1999 cpm and negative for time spent ≥4000 cpm in younger children, whereas associations were negative for time spent in 0–99 cpm, positive for time spent in 100–3999 cpm and negative for time spent ≥11,000 cpm in older children.

## 5 Discussion

In this paper, we determined associations for the entire triaxial PA intensity spectrum with BMI and WHtR in a large sample of preschoolers. Our findings show that associations between PA, BMI, and WHtR are evident already at a young age. While associations at the lower end of the intensity spectrum (vertical axis) were similar for BMI and WHtR (i.e., negative for time spent in 0–99 cpm and positive for time spent in 100–2999 cpm), associations for higher intensities were evident only for WHtR (i.e., negative for time spent in ≥5000 cpm). The associations at the lower end of the intensity spectrum were consistent across sex and age groups, whereas associations for higher intensities were less consistent. Associations were stronger for boys than for girls but comparable for younger and older children.

Aadland et al. (2021) determined the multivariate association pattern between the PA intensity spectrum and BMI in 1182 preschoolers and found a negative association for time spent in SED (0–99 cpm), positive associations for time spent in LPA and partly MPA (100–2999 cpm), and negative associations for time spent in VPA (≥4000 cpm). Thus, in the lower end of the intensity spectrum, *less* time spent sedentary, and *more* time spent in low to moderate intensities were associated with *higher* BMI. These findings were consistent with the findings by Wiersma et al. (2020), who observed a negative association between SED and BMI (7 studies) and a tendency of a positive association between LPA and weight status (6 studies) in a meta-analysis of studies in young children. In the present study, using the same analytic approach as Aadland et al. (2021), we confirm these associations in another large sample of preschoolers. These associations were consistent across sex and age groups and for both BMI and WHtR. Thus, it seems like preschoolers with higher BMI and WHtR sit less and move more than their peers having a lower BMI and WHtR. On the contrary, using similar methodology, previous studies have shown an expected unfavorable association for SED and then gradually increased favorable associations for higher intensities, particularly VPA, with motor skills in preschoolers (Nilsen et al., 2020) and metabolic health indices in schoolchildren (Aadland et al., 2018, Aadland et al., 2020, Aadland et al., 2019). Consistent with the view of Wiersma et al (Wiersma et al., 2020), we suggest the associations for SED and LPA with BMI may be a result of the inability of BMI to capture adiposity as it is well known that BMI cannot separate lean and fat mass. Thus, a higher BMI among preschoolers probably reflect early growth and development to a larger extent than adiposity, which is consistent with increased BMI throughout childhood (Cole et al., 2000) and increased PA level in children from 2 to 6 years (Schmutz et al., 2018, Nilsen et al., 2019). Although we used a SED cut point of 100 cpm as recommended in the literature (Ridgers et al., 2012, Kim et al., 2014), differential misclassification of SED and LPA by maturational status could be an alternative explanation of our findings. Moreover, epoch length substantially affect time spent in SED and LPA in young children, but associations for SED and LPA with BMI have been shown to be marginally affected by epoch length (Aadland and Nilsen, 2022). Future studies should investigate age-specific association patterns between the PA intensity spectrum, BMI, and WHtR in large samples throughout childhood to further clarify the relationship with age and possibly verify our findings.

Associations for higher PA intensities with BMI are less consistent across studies. Wiersma et al. (2020) found a positive association between MVPA and BMI in boys, a tendency for a positive association between VPA and BMI, negative associations for both VPA and MVPA with weight status, and no associations between PA and waist circumference in their meta-analysis. Aadland et al. (2021) found a negative association between VPA (i.e., intensities >4000 cpm) and BMI, but this association was only observed in older children (4.8–6.5 years) and was somewhat stronger in girls than in boys. In the present study, based on a similarly sized sample of children in the same age group and using similar methodology, we could not verify these findings. We found no significant associations between intensities >3000 cpm and BMI in the total sample, no differences between younger and older children, but negative associations for intensities >4000 cpm in boys. We have no explanation for the discrepancies between the present findings and those by Aadland et al. (2021) beyond highlighting that associations are very weak and that small sampling differences may alter findings. In Aadland et al (Aadland et al., 2021), the strongest standardized association between vigorous intensities and BMI was −0.09, compared to −0.03 in the present study, and −0.12 between VPA and weight status found by Wiersma et al (Wiersma et al., 2020). Interestingly, the association of −0.09 between vigorous intensities and WHtR in the present study was identical to the association shown for BMI previously (Aadland et al., 2021). The tendencies for more positive associations for MVPA and VPA with BMI found by Wiersma et al (Wiersma et al., 2020) than in the present study and the study by Aadland et al (Aadland et al., 2021), could be a result of relying on studies with longer epoch. Aadland et al (Aadland and Nilsen, 2022) compared association patterns for the PA intensity spectrum with BMI using different epoch lengths and found a change from negative to positive associations for vigorous intensities when epoch length increased. The marginally stronger association for WHtR than for BMI in the present study suggests that WHtR might marginally better capture adiposity resulting from inactivity than BMI or, alternatively, that a higher WHtR is a marginally stronger predisposing factor for inactivity than higher BMI. Nevertheless, these findings together suggest that weak negative associations between VPA, BMI, WHtR, and weight status are evident in 3–5-year-old children.

Standardized associations for intensity-specific PA with BMI or weight status found by Wiersma et al (Wiersma et al., 2020) varied from −0.12 to 0.13. The strongest association observed by Aadland et al (Aadland et al., 2021) were 0.13 (BMI), whereas the strongest association observed in the present study was 0.17 (BMI and WHtR), all observed for LPA. These findings show that large samples are needed to arrive at sound conclusions when investigating these association patterns in young children. Such weak associations also mean that subgroup analyses are challenging due to reduced power and increased instability of findings. Aadland et al (Aadland et al., 2021) found stronger associations in older than in younger children and somewhat stronger associations in girls than in boys. In the present study, we found rather similar associations for younger and older children and stronger associations in boys than in girls. These inconsistent findings clearly illustrate that caution is needed when drawing conclusions from original studies and that knowledge synthesis from systematic reviews and meta-analyses are needed, though their conclusions are hampered by lack of harmonization among original studies, particularly with respect to data reduction and analytic approaches for accelerometry data.

## 6 Strengths and limitations

The prevailing evidence on associations between PA, BMI, and WHtR is limited by small sample sizes, the selection of few intensity variables, and a lack of harmonization of analytical approaches for handling of accelerometry data (Wiersma et al., 2020). Strengths of this study are the large sample size and an analytic approach allowing us to determine associations for the entire PA intensity spectrum from triaxial accelerometry. Since multivariate pattern analysis can handle completely multicollinear explanatory variables (Wold et al., 1984), it allows for determination of detailed association patterns of the entire PA intensity spectrum with outcomes. Using a high-resolution triaxial intensity spectrum, we circumvent the well-known “cut point conundrum”, as we did not need pre-determined intensity cut points. Importantly, the triaxial intensity spectrum provided information that was not captured by the vertical axis or the vector magnitude intensity spectra, which is consistent with previous findings (Aadland et al., 2021, Aadland et al., 2019). However, we have focused our interpretation on the vertical axis because there is limited knowledge of which activities and intensities that are captured across the anteroposterior and the mediolateral axes. Moreover, we interpreted intensities according to the cut points suggested by Evenson et al (Evenson et al., 2008). Nevertheless, the reporting of association patterns across the entire range of intensities for all axes allows for alternative interpretations post hoc. More research is needed to improve our understanding of which activities and intensities that are captured across the intensity spectrum of the triaxial intensity spectra to inform interpretation of our findings and inform PA guideline development.

The cross-sectional design of the present study restricts conclusions regarding causality of the observed associations. While PA can induce weight loss, observational evidence in children suggest that adiposity may be a stronger determinant of future PA than vice versa (Metcalf et al., 2011, Hjorth et al., 2014). Thus, longitudinal studies are needed to investigate the temporality of the associations between PA, BMI, and WHtR, as well as other outcomes that better capture adiposity, during childhood.

## 7 Conclusion

We determined the multivariate association patterns for PA with BMI and WHtR in a large sample of preschool children. Higher BMI and WHtR were associated with less time spent sedentary and more time spent at lower intensities. Higher WHtR, but not BMI, was associated with less time spent in vigorous intensity. Some differences were observed between sex and age groups, but these findings should be interpreted carefully. Our findings suggest public health initiatives should be initiated during the early years to have optimal conditions of succeeding in prevention of unhealthy weight gain and in promotion of optimal PA trajectories during childhood.

## CRediT authorship contribution statement

**Eivind Aadland:** Conceptualization, Methodology, Software, Formal analysis, Investigation, Visualization, Supervision, Project administration, Funding acquisition, Writing – original draft, Writing – review & editing. **Ada Kristine Ofrim Nilsen:** Methodology, Data curation, Investigation, Supervision, Project administration, Writing – review & editing. **Elisabeth Straume Haugland:** Investigation, Writing – review & editing. **Kristoffer Buene Vabø:** Investigation, Writing – review & editing. **Katrine Nyvoll Aadland:** Methodology, Investigation, Supervision, Project administration, Funding acquisition, Writing – review & editing.

## Declaration of Competing Interest

The authors declare that they have no known competing financial interests or personal relationships that could have appeared to influence the work reported in this paper.
