# Supplementary figures and images for: The multivariate physical activity signatures associated with body mass index and waist-to-height ratio in 3–5-year-old Norwegian children
Source: Prev Med Rep. 2022 Jul 29;29:101930. doi: 10.1016/j.pmedr.2022.101930 (PMC9356261; doi:10.1016/j.pmedr.2022.101930)

Multivariate correlation coefficient

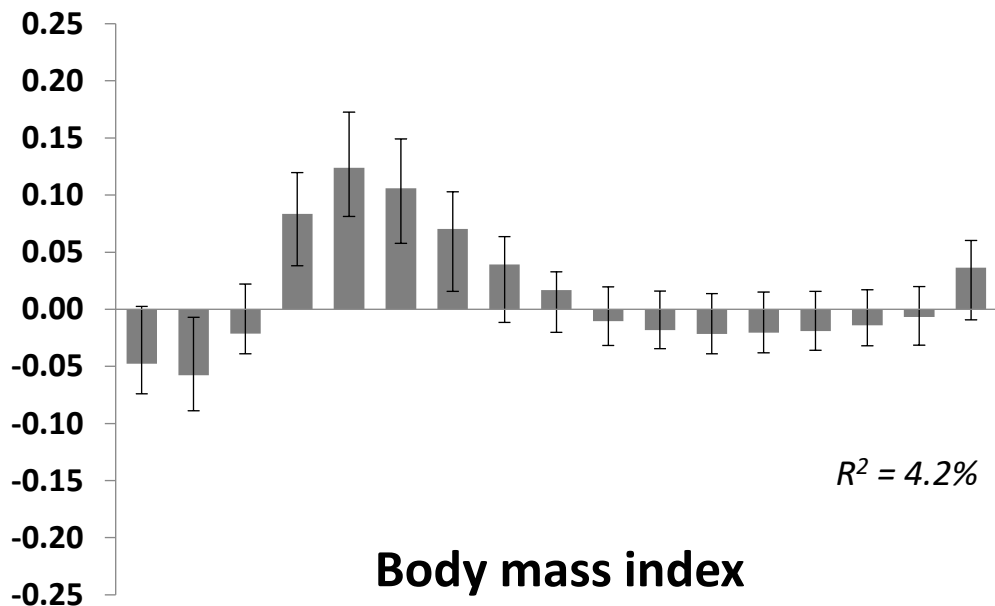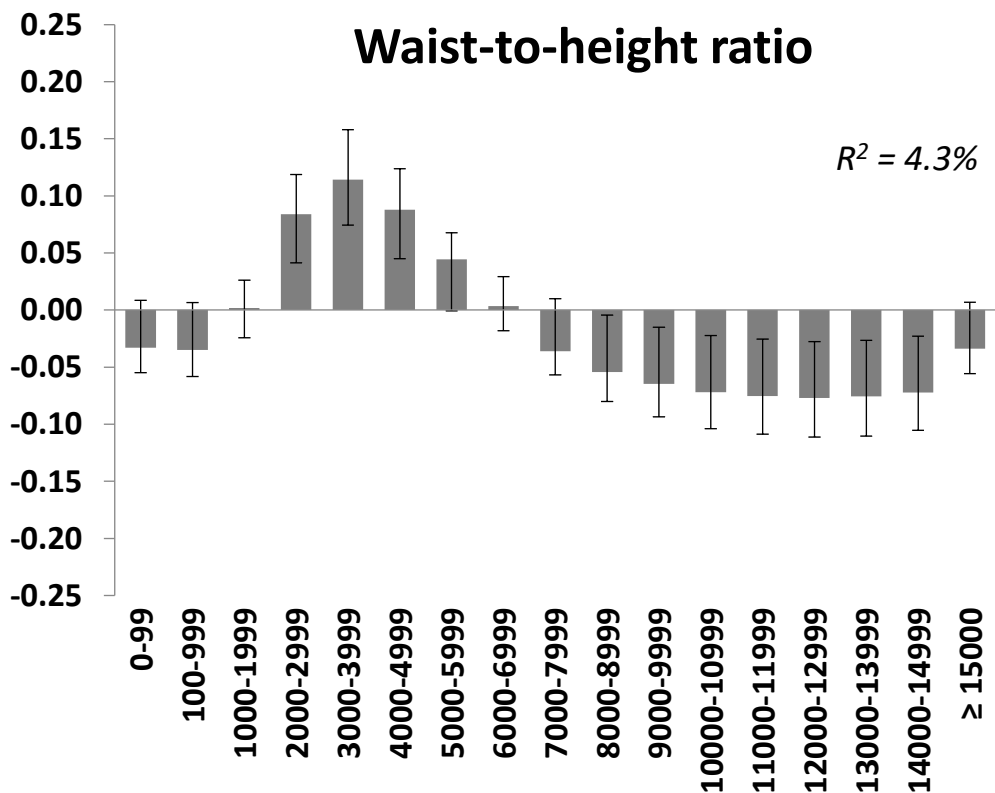

Physical activity intensity (counts per minute)

Supplement: Supplementary data 1 [file mmc1.pdf]
